# COVID-19 and Preeclampsia: A Systematic Review of Pathophysiological Interactions

**DOI:** 10.1055/s-0043-1770091

**Published:** 2023-07-21

**Authors:** Maria Isabel do Nascimento, Alfredo de Almeida Cunha, Nercélio Falcão Rangel Netto, Raphael Alves dos Santos, Rodrigo Roberto Barroso, Thiago Rodrigues de Carvalho Alves, Wender Emiliano Soares

**Affiliations:** 1 Faculdade de Medicina, Universidade Federal Fluminense, Niterói, RJ, Brazil; 2 Faculdade de Medicina, Universidade do Estado do Rio de Janeiro, Rio de Janeiro RJ, Brazil; 3 Faculdade de Medicina, Universidade Federal do Rio de Janeiro, Rio de Janeiro, RJ, Brazil

**Keywords:** COVID-19, SARS-CoV-2, Pré-eclâmpsia, Eclâmpsia, Patogênese, COVID-19, SARS-CoV-2, Preeclampsia, Eclampsia, Pathogenesis

## Abstract

**Objective** : To review the literature and synthesize evidence on pathophysiological interactions attributed to the simultaneous occurrence of COVID-19 and preeclampsia.

**Methods** : A systematic review was conducted from November (2021) to January (2022) to retrieve observational studies published on the PubMed, LILACS, SciELO Brazil and Google Scholar databases. The search was based on the descriptors [(eclampsia OR preeclampsia) AND (COVID-19)]. Quantitative studies that pointed to pathophysiological interactions were included. Literature reviews, studies with HIV participants, or with clinical approach only were excluded. The selection of studies was standardized and the evaluation was performed by pairs of researchers.

**Results:** In this review, 155 publications were retrieved; 16 met the inclusion criteria. In summary, the physiological expression of angiotensin-converting enzyme-2 (ACE-2) receptors is physiologically increased in pregnant women, especially at the placental site. Studies suggest that the coronavirus binds to ACE-2 to enter the human cell, causing deregulation of the renin-angiotensin-aldosterone system and in the ratio between angiotensin-II and angiotensin-1-7, inducing manifestations suggestive of preeclampsia. Furthermore, the cytokine storm leads to endothelial dysfunction, vasculopathy and thrombus formation, also present in preeclampsia.

**Conclusion:** The studies retrieved in this review suggest that there is a possible overlap of pathophysiological interactions between COVID-19 and preeclampsia, which mainly involve ACE-2 and endothelial dysfunction. Given that preeclampsia courses with progressive clinical and laboratory alterations, a highly quality prenatal care may be able to detect specific clinical and laboratory parameters to differentiate a true preeclampsia superimposed by covid-19, as well as cases with hypertensive manifestations resulting from viral infection.

## Resumo

**Objetivo** : Revisar a literatura e sintetizar evidências sobre interações fisiopatológicas atribuídas à ocorrência simultânea de COVID-19 e pré-eclâmpsia.

**Métodos** : Uma revisão sistemática foi conduzida entre novembro (2021) a janeiro (2022) para recuperar estudos observacionais publicados no PubMed, LILACS, SciELO Brasil e Google scholar. A busca foi baseada nos descritores [(eclâmpsia OR pré-eclâmpsia) AND (COVID-19)]. Estudos quantitativos que apontaram interações fisiopatológicas foram incluídos. Estudos de revisão, com participante HIV e apenas com enfoque clínico foram excluídos. A seleção dos estudos foi padronizada com avaliação por duplas de pesquisadores.

**Resultados:** Nesta revisão, 155 publicações foram recuperadas; 16 preencheram os critérios de inclusão. Em síntese, a expressão fisiológica de receptores da enzima conversora da angiotensina-2 (ECA-2) é fisiologicamente potencializada em gestantes, especialmente no sítio placentário. Os estudos sugerem que o coronavírus se liga à ECA-2 para entrar na célula humana, ocasionando desregulação do sistema renina-angiotensina-aldosterona e da razão entre angiotensina-II e angiotensina-1-7, induzindo manifestações sugestivas de pré-eclâmpsia. Ademais, a tempestade de citocinas conduz à disfunção endotelial, vasculopatia e formação de trombos, também presentes na pré-eclâmpsia.

**Conclusão:** Os estudos recuperados nesta revisão sugerem que a superposição de alterações fisiopatológicas entre a COVID-19 e a pré-eclâmpsia envolve, principalmente, a ECA-2 e disfunção endotelial. Tendo em vista que a pré-eclâmpsia cursa com alterações clínicas e laboratoriais progressivas, a atenção pré-natal de qualidade pode ser capaz de detectar parâmetros clínicos e laboratoriais importantes para diferenciar a pré-eclâmpsia verdadeira sobreposta por COVID-19, bem como os casos que mimetizam a doença hipertensiva consequente à infecção viral.

## Introduction

The concurrence of pregnancy-related diseases (gestational hypertension, preeclampsia and eclampsia) with the COVID-19 virus is a clinical novelty, classified as a serious maternal risk. 1 2 A living systematic review with meta-analysis in pregnant and recently pregnant women reported severe COVID-19 infection in 9%; the intensive care unit admission required in 4%; invasive ventilation used in 2%; and extracorporeal membrane oxygenation administered in 0.2%. 3

Preeclampsia is characterized by new onset hypertensive manifestations occurring with or without proteinuria in the last half of pregnancy or postpartum. In general, its onset occurs after 20 weeks of gestation or earlier in the presence of gestational trophoblastic disease or hydrops fetalis, and normal physiological pressure levels return within 12 weeks after the birth of the conceptus. 4

The pathophysiology of preeclampsia is still knowledge under construction. Theoretically, there is a failure in the complete remodeling of the maternal spiral arteries that are meant to guarantee adequate blood flow. The impairment conversion from small and higher resistance arterioles into large arteries leads to high resistance of blood flow, hypoperfusion, and hypoxemia, culminating in maternal systemic endothelial cell dysfunction. 4

Hypertensive disorders of pregnancy affect approximately 10% of pregnant women worldwide, an estimate that includes preeclampsia and eclampsia, gestational hypertension and chronic hypertension. 5 A meta-analysis that included 10 studies and 2988 women estimated the prevalence of preeclampsia in 6.7% with 95% Confidence interval (CI) of 4.9%-8.6%, in Brazil. 6

A meta-analysis that combined data of preeclampsia in pregnant women infected with the new coronavirus from 10 studies estimated a prevalence of 8.2% (95% CI: 5.7 to 11.7%). In addition, stratification by country showed prevalence of 10.8% (95% CI: 8.1% to 14.3%) and 10.4% (95% CI: 5.0% to 20.1%) for the United States and China, respectively. 7 The concurrence of COVID-19 with preeclampsia has gained popularity in the literature. The possibility that SARS-CoV-2 infection contributes to the development of preeclampsia has been raised, but there is no consensus. For example, the lack of positivity of preeclampsia-specific anti-angiogenic and angiogenic markers has directed the explanatory reasoning more towards COVID-19 than placental disorders. 8

Given the severity of the overlap between COVID-19 and preeclampsia, it is imperative to deepen the knowledge of the pathophysiological interactions, which provides information to support clinical approaches and stimulate the development of future investigations. The aim of this review was to present a narrative synthesis on the pathophysiological interactions attributed to the simultaneous presence of COVID-19 and preeclampsia.

## Methods

This study is a narrative synthesis systematic review in line with Popay et al. (2006) 9 which was conducted to answer the research question; “What are the pathophysiological interactions determined by the simultaneous presence of COVID-19 and preeclampsia?”, which was structured according to the PICO strategy for formulating a research question; P (population/medical condition): (pregnancy and preeclampsia); I (intervention/exposure): COVID-19; C (comparison): not applicable; O (outcome/outcome): not applicable. The review was structured according to the criteria established by *Preferred Reporting Items for Systematic Reviews and Meta-Analyses* (PRISMA). 10

## Search Strategy

The following bibliographic databases were used to retrieve the publications of interest; (i) Medical Literature Analysis and Retrieval System Online (PubMed/Medline), (ii) Scientific Electronic Library Online (SciELO) and (iii) Latin American and Caribbean Health Sciences Literature (LILACS). The Health Sciences Descriptors that were used to build the search strategy were obtained from the Health Sciences Descriptors database; DeCS/MeSH terms for both English and Portuguese. The combinations of terms and boolean operators that guided the search in the Medline/PUBMED database were (Pregnancy AND Preeclampsia OR Eclampsia) AND (COVID-19). In LILACS and SciELO Brasil, the search considered only COVID-19 OR SARS-Cov-2 AND Preeclampsia OR Eclampsia, which were in Portuguese.

## Manual Search

An additional broader search strategy was carried out aimed at retrieving information that may have been omitted or not captured by the abovementioned search strategies. This step included consulting the first 200 Google Scholar records, and selecting the relevant literature for full review. In addition, the reference lists of the selected articles were also scanned to obtain articles for full reading.

## Inclusion and Exclusion Criteria

The simultaneous manifestation of COVID-19 and preeclampsia in the gestational period (does not include puerperium) was the inclusion criterion in the review. The Exclusion criteria were: (i) review studies (ii), studies with simultaneous Human Immunodeficiency Virus (HIV) infection, (iii) language other than English, Portuguese and Spanish, (iv) studies with an exclusive clinical approach without pathophysiological aspects/ approach. This last criterion actually excluded clinical and/or epidemiological studies that although dealing with clinical aspects and related outcomes, did not discuss the possible pathophysiological mechanisms manifested from the interaction of COVID-19 and preeclampsia in a patient having both conditions simultaneously.

## Data Collection

The selection of studies was carried out by pairs of researchers, performed independently and in a standardized way. Discrepancies were resolved with the participation of a third researcher. The data collection included the characteristics of the studies (authors, year, journal, title, country), the population studied (sample size, age of women, gestational age) and possible pathophysiological interactions.

## Results

**Fig. 1:**
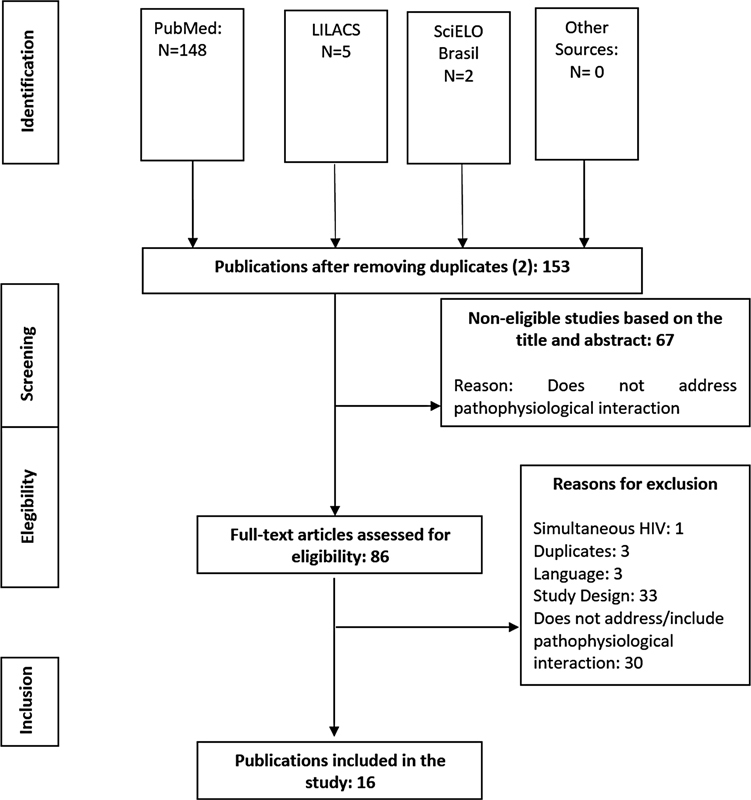
Flowchart of the steps that were followed during the selection and inclusion of publications.

This review retrieved 155 publications from the searches performed in the bibliographic databases of Medical Literature Analysis and Retrieval System Online (PubMed/Medline) (n = 148), Latin American and Caribbean Literature in Health Sciences (LILACS) (n = 5), Scientific Electronic Library Online (SCIELO BRASIL) (n = 2) and other sources (n = 0). After excluding the duplicates (2), inappropriate publications (67) based on the content of the title and abstract were discarded. Of the articles eligible (n = 86) for full text reading, 70 were excluded for the following reasons: HIV infection (n = 1), duplicate (n = 3), study design (n = 33), language (n = 3) and lack of information/approach regarding pathophysiology and interaction (n = 30). Finally,16 studies 11 12 13 14 15 16 17 18 19 20 21 22 23 24 25 26 were effectively included in this review ( Fig. 1 ).

The included studies were conducted in the United States (n = 4), Spain (n = 2) and Canada (n = 2). The other studies (n = 8) are from 7 different countries of which one is of multicentric/international origin. Five of the studies are case reports which presented the clinical evolution of pregnant women affected by COVID-19 with symptoms suggestive of preeclampsia. Among the most robust evidence, a case-control study evaluated hypertensive disorders of pregnancy by comparing 173 pregnant women with COVID-19 to 733 with a negative SARS-CoV-2 test and found a higher frequency of gestational hypertension, preeclampsia and preeclampsia with severity features in the positive COVID-19 group in contrast to the negative COVID-19 group. 23 Chart 1 summarizes the characteristics of the studies included.

**Chart 1:** Characteristics of the studies included in the systematic review

Author/Year	Country	Study design	Type of participant	Participant (n)	Other assessments
Naeh et al. (2022) 11	Canada	Case Report	Pregnant Women	1	X
Aydin et al. (2021) 12	Turkey	Retrospective Study	Pregnant Women	167	X
Laresgoiti-Servitje et al. (2021) 13	Mexico	Case-Control	Pregnant Women	298 +/828-	X
Osaikhuwuomwan et al. (2021) 14	Nigeria	Descriptive	Pregnant Women	19 +/48-	X
Verma et al. (2021) 15	USA	Descriptive Translational	Pregnant Women	5 +/5-	Placenta
Federici et al. (2020) 16	France	Case Report	Pregnant Women	1+	X
Ahmed et al. (2020) 17	UK	Case Report	Pregnant Women	1+	X
Baracy et al. (2021) 18	USA	Historical Cohort	Pregnant Women	70 +/210-	X
Shchegolev et al. (2021) 19	Russia	Comparative Morphological Study	x	23 +/7-	Placenta
Papageorghiou et al. (2021) 20	Multinational	Prospective Observational	Pregnant Women	725 +/1459-	X
Lu-Culligan et al. (2021) 21	USA	Obervational	Pregnant Women	15+	Placenta
Braga and Sass (2020) 22	Brazil	Case Report	Pregnant Women	1+	X
Bloise et al. (2021) 23	Canada	Cross-Sectional Study	Pregnant Women	87+	Placenta
Shanes et al. (2020) 24	USA	Comparative	Pregnant Women	16+	Placenta
Garcia Rodriguez et al. (2020) 25	Spain	Case Report	Pregnant Women	1+	X
Mendoza et al. (2020) 26	Spain	Observational	Pregnant Women	42+	X

X: no additional evaluation was performed. +: number of positive COVID-19 cases. –: number of negative COVID-19 cases.

The selected studies provided key contributions regarding the pathophysiological mechanisms resulting from SARS-CoV-2 infection and the changes in the placental site found in preeclampsia. The role of the Angiotensin-Converting Enzyme 2 (ACE2) was highlighted in most studies. On the other hand, uncertainties were supported by arguments that women diagnosed with preeclampsia or those with higher risk factors were more frequently submitted to SARS-CoV-2 tests, and therefore had confirmed infection. 20 These authors acknowledged that the examination of the placenta could have helped to determine the extent of vasculitis in relation to the severity of infection, and contributed to the understanding of the pathophysiological interaction, a fact that was not contemplated in their study ( Chart 2 ).

**Chart 2:** Pathophysiological interactions highlighted in COVID-19 and preeclampsia studies

Author	Main pathophysiological findings or conclusions
Naeh et al. 11	“Endothelial dysfunction has been suggested as the mechanism for both manifestations; [...] hypertension and kidney injury ...”
Aydin et al. 12	“Upon entry into the human body, SARS-CoV-2 spike binds to ACE2 receptor through its receptor-binding domain. ... we found a significant difference in the laboratory parameters among the groups. D-dimer is an indicator of fibrinolysis and plays a key role in the diagnosis of thromboembolism.”
Laresgoiti-Servitje et al. 13	“…we further explored the placentas of a group of pregnant women regarding the presence of placental dysmaturity, vasculopathy, fibrinoid, chorangiosis, chorioamnionitis, hemorrhage, or infarction. The placentas of women infected with SARS-CoV-2 had a higher rate of fibrinoid deposition, a clinical feature of maternal vascular malperfusion, than controls”.
Osaikhuwuomwan et al. 14	“…adverse pregnancy outcomes were high, especially among those with other co-morbidities such as pre-eclampsia or other complications because respiratory syndromes may aggravate pulmonary oedema and decrease oxygen saturation. The endothelial dysfunction associated with pre-eclampsia predisposes patients to respiratory failure from pulmonary oedema.”
Verma et al. 15	“In sum, we demonstrate that SARS-CoV-2 colonizes fetal trophoblasts, stromal cells, and macrophages in the placenta, which express the ACE2 receptor. S binding to ACE2 leads to reduction of the receptor expression and results in alterations of the RAS pathway—changes that are similar to those typically noted in pre-eclampsia.”
Federici et al. 16	“HELLP is an acronym which refers to the triad of microangiopathic haemolysis with elevated liver enzymes and a low platelet count. HELLP syndrome is a serious complication of pre-eclampsia [...]. Some biological disorders linked to SARS-CoV-2 infection associated with hypertension may mimic a pre-eclampsia or a HELLP syndrome.”
Ahmed et al. 17	“Both pre-eclampsia and COVID-19 infection are examples of microvascular disease causing endothelial injury. They both cause a high prothrombotic tendency leading to multiorgan failure. The presence of both diseases likely had either a synergistic or an opportunistic effect, which may have led to severe clinical manifestations via the interplay of the renin-angiotensinogen-aldosterone system in their pathogenesis.”
Baracy et al. 18	“When comparing only COVID-19 positive pregnancies, early infection conferred a significantly higher risk for HDP than late infection. This observation is consistent with the inflammatory explanation of increased HDP risk in COVID-19. Through modulation of angiogenic factors and inflammatory cytokines, it is likely that COVID-19 exerts maximal impact on placental physiology at earlier gestations, enabling these physiologic changes to manifest as HDP over time.”
Shchegolev et al. 19	“...study demonstrated increased level of VEGF expression mainly in syncytiotrophoblast of the terminal villi in parturient women with moderate COVID-19 to a greater extent than in women with mild disease severity. These changes, along with increased number of syncytial knots, also indicate the development of placental hypoxia in parturient women with COVID-19. hypoxia promotes increased production of pro-angiogenic factors by placental cells, in particular, VEGF, which not only regulates proliferation and migration of endotheliocytes, but also contributes to BP elevation in pregnant women.”
Papageorghiou et al. 20	“…pre-eclampsia and GH are vascular conditions, preceding infection with SARS-CoV-2, which increase the risk for COVID-19 in the same way essential hypertension does.”
Lu-Culligan et al. 21	“…we found that ACE2 protein was present at significantly higher levels in term placentas collected from COVID-19 cases. These findings suggest that detection of ACE2 mRNA expression is not a reliable surrogate for ACE2 protein expression in the placenta and, importantly, that ACE2-mediated risk for placental infection by SARS-CoV-2 may vary over the course of pregnancy, with our detection of higher ACE2 levels in the first and second trimesters suggesting that the most vulnerability may exist prior to term.”
Braga and Sass 22	“Thrombocytopenia in patients with COVID-19 appears to be multifactorial, including endothelial damage, platelet activation with aggregation and thrombosis, impairment of bone marrow and megakaryocyte activity. [...] synergism of these pathophysiological mechanisms could accelerate the compromise of maternal conditions”
Bloise et al. 23	“...SARS-CoV-2 present in the maternal circulation has the potential to enter the maternal blood bathed syncytiotrophoblast and infect the placenta via ACE2 binding.”
Shanes et al. 24	“The histologic changes of MVM are thought to represent some chronicity, though exact timing is unknown, and these features can be seen in women who develop pre-eclampsia only during or after childbirth. Whether systemic vascular changes due to maternal COVID-19 are responsible for the histologic changes of MVM cannot be determined. [...] there are increased rates of maternal vascular malperfusion features and intervillous thrombi, suggesting a common theme of abnormal maternal circulation, as well as an increased incidence of chorangiosis”
Garcia Rodriguez et al. 25	“…we believe SARS-COV-2 infection could promote brain endothelial damage, triggering the cited neurological complications in our patient.”
Mendoza et al. 26	“…this is the first study to describe the incidence of signs and symptoms of PE in a relatively large cohort of pregnancies with COVID-19 and to show that a PE-like syndrome could be induced by severe COVID-19. [...] Several disorders have previously proved to imitate PE since they share some of the clinical and laboratory findings of patients with PE. The pathophysiologic causes of these conditions include vasospasm, platelet activation or destruction, microvascular thrombosis, endothelial cell dysfunction, and reduced tissue perfusion”.

In addition to the pathophysiological findings revealed from the evaluation of pregnant women, additional information was extracted from five studies that analyzed the placentas. 15 19 21 23 24 The virus colonization was more abundant in maternal decidua and fetal villous tissue, showing an important inflammatory process with leukocyte infiltration. In addition, there was greater severity in the preterm placenta, with subsequent implication in the expression of ACE2. 15 The expression of genes involved in the entry of SARS-CoV-2 into placental cells appears to be downregulated as pregnancy progresses, thus suggesting greater vulnerability to infection in the first trimester. 23 Syncytial knots in the villi and intervillous bridges indicate the presence of a pre-placental hypoxic state. 19 Marked expression of Vascular Endothelial Growth Factor (VEGF) in the capillary endotheliocytes and syncytiotrophoblast were detected in placental morphology, contributing to the suspicion of preeclampsia like syndrome. 19 Compared to placentas from controls which had melanoma, a placenta with maternal vascular malperfusion was a statistically significant finding, accompanied by decidual arteriopathy, fibrinoid necrosis of maternal vessels, and mural hypertrophy of the membrane arterioles. 24

## Discussion

The present review consists of a narrative synthesis on the pathophysiological interactions of COVID-19 and preeclampsia reported in primary studies. Despite the gaps that persist, studies highlighted the role of biological mechanisms resulting from the binding of SARS-CoV-2 with angiotensin-converting enzyme-2 (ACE2) receptors and the hypertensive manifestations subsequent to the vasoconstrictor effects. The literature consistently recognizes the overlap of pathophysiological mechanisms common to preeclampsia and COVID-19, highlighting endothelial dysfunction, its inflammatory effects and thrombogenic manifestations.

The increased expression of ACE2 receptors at the placental site in cells promoting blood flow remodeling and trophoblast invasion is a physiological condition, but this seems to make pregnant women more prone to the development of severe COVID-19. 18 To enter the cell, the virus promotes the binding of its spike protein (Spike-like protein) to the ACE2 receptors, reducing the bioavailability of the enzyme. Thus, there is a picture of vasoconstriction without the counterbalance expression of Angiotensin 1-7 responsible for vasodilation and a consequent decrease in blood pressure. 27

In addition to the vasodilatory effect, Angiotensin 1-7 also has anti-inflammatory and antithrombogenic activity. 27 However, an investigation of the levels of D-dimer, prothrombin time and International Normalized Ratio (INR) in 167 women with COVID-19 classified into 3 groups defined by gestational age detected lower measurements of prothrombin time and INR in the group ≥ 24 weeks, but higher fractions of D-dimer. 12 Although the study included 20 women with preeclampsia and 2 with hemolysis, elevated liver enzymes and low platelets (HELLP) syndrome, the authors considered their findings as physiological because the group ≥ 24 weeks also included postpartum women. 12

The fetus determines immunological reactions that require compensatory mechanisms capable of harmoniously leading the pregnancy to term, with decidual natural killer cells playing a key role in placentation and in the maintenance of a healthy pregnancy. 4 The immune function that natural killer cells perform in the peripheral blood differs from the remodeling action they perform in the decidua. 4 In the decidual compartment, they work with the trophoblast to produce uterine vascular remodeling, to the detriment of the naturally expected cytotoxic activity. The SARS-CoV-2 infection disrupts this immunologic harmony and can negatively impact obstetric and perinatal outcomes. 4 Infection by SARS-CoV-2 leads to a decrease in the concentration of this type of cell in peripheral blood since they are mobilized to fight the virus and not directed to the decidua. The reduction in migration culminates into a reduction in the population of natural killer cells in the decidua, which may cause poor placental perfusion, one of the possible etiologies of preeclampsia. 4

There is a growing body of knowledge regarding the placenta investigating the relationship between SARS-CoV-2 infection and the disruption of the renin-angiotensin system on pregnancy outcomes. 28 29 The infected placentas indicated that the virus was found in multiple compartments of the maternal-fetal interface, including trophoblasts, stromal, immune system, and epithelial cells. 15 In addition, the study reported inflammatory infiltrates in SARS-CoV-2-infected preterm placentas while non-infected preterm placentas had minor or no inflammation. The authors also found a reduction in the expression of ACE2, which leads to an imbalance in the renin-angiotensin system, as well as an increased expression of pro-inflammatory substances accompanied by a simultaneous suppression of the protective arm of the renin-angiotensin system. The disruption of the normal physiological expression of the angiogenic and antiangiogenic factors determined by the ratio of placental growth factor [PIGF] to soluble fms-like tyrosine kinase-1[sFIt-1] substantiates the mechanism underlying the endothelial dysfunction present in multiple organ failure. 15

Although some studies point to an association between COVID-19 and preeclampsia/eclampsia/HELLP syndrome, uncertainties still remain. It is possible that the hypertensive condition constitutes a preeclampsia-like syndrome. 30 Mendoza et al. (2020) 26 investigated the existence of a possible relationship between COVID-19 and preeclampsia by comparing pregnant women with COVID-19 which were categorized as severe (n = 8) and non-severe infection (n = 34). The results indicated that in pregnant women with severe infection, six of them had symptoms suggestive of preeclampsia, but markers of placental disease were only found in one. In view of this situation, the authors recommend caution in the diagnosis of preeclampsia and consider the possibility that pregnant women with COVID-19 may experience a preeclampsia-like syndrome. The analysis of markers such as the uterine artery pulsatility index (UtAPI) and the evaluation of antiangiogenic and angiogenic factors (sFlt-1/ PIGF) are crucial in these cases to differentiate the two conditions. 26 Although the distinction between true preeclampsia and preeclampsia-like syndrome resulting from COVID-19 is not straightforward, efforts must be made to characterize each of these conditions in order to avoid interventions and induction of unnecessary childbirth. 12 31

Another condition that shares similar pathophysiological interactions with preeclampsia and SARS-CoV-2 infection is posterior reversible encephalopathy syndrome (PRES). Although no single mechanism explains the development of PRES, vascular hyperperfusion seems to play an important role when blood hypertensive spikes (sudden high blood pressure) are present and the brain autoregulation process is ineffective, leading to hyperperfusion with extravasation of plasma and macromolecules into brain tissue. 32 Endothelial dysfunction with cytokine release is also prominent in PRES and may be exacerbated by the presence of COVID-19 toxins. 33 The adaptation of the immune system to physiologically accommodate the gestational period is interrupted by viral infection and exacerbated by the concurrence of placental disease and cerebral hyperperfusion. Although establishing the clinical boundaries of the three entities is a challenge, the simultaneous presence of the 3 conditions must be seen as a possible reality during these times of the pandemic.

This study sought to synthesize the pathophysiological mechanisms and interactions that underlie co-occurrence of SARS-CoV-2 infection and preeclampsia, and to improve the understanding of these two entities as well as stimulate the development of studies that investigate this problem. However, this study presented some limitations. At first, the inclusion criteria based on primary quantitative studies may have omitted publications that could possibly help in understanding the problem. To this effect, we employed a combination of designs ranging from case reports to prospective cohorts to minimize or offset this deficiency, providing a wide possibility of representative contributions from specific contexts that used different methodological approaches. Another limitation stems from the language restriction, since the review only included studies written in English, Portuguese and Spanish.

## Conclusion

Understanding the risk that the coronavirus poses to pregnant women proved to be vital in these times of the pandemic. In summary, as one of the components of the renin-angiotensin-aldosterone system, the expression of ACE2 receptors is increased in pregnant women, especially at the placental site. The coronavirus binds to ACE2 receptors as part of the mechanism of entry into the human cells and this leads to the deregulation of the system and in the ratio between angiotensin-II and angiotensin1-7, thus imitating and/or potentiating the picture of preeclampsia. Furthermore, the cytokine storm leads to endothelial dysfunction and thrombus formation, which is also classically present in preeclampsia. Given that preeclampsia courses with progressive clinical and laboratory alterations, a highly quality prenatal care may be able to detect specific clinical and laboratory parameters to differentiate a true preeclampsia superimposed by covid-19, as well as cases with hypertensive manifestations resulting from viral infection. The collective message or consensus arising from the studies in this narrative synthesis is that there is a possible overlap of pathophysiological alterations between COVID-19 and preeclampsia. In view of the importance of ACE2 in maintaining physiological blood pressure levels and the role it plays in the SARS-CoV-2 infectious process, establishing a characteristic pathophysiological distinction between infectious disease and placental disease is a major challenge. Given the complexity of the topic and the merely synthetic narrative purpose that guided this review, it is necessary and recommended that more robust investigations aimed at deepening the knowledge about the pathophysiological interactions of these two important nosological entities should be performed.
